# Facets of trait impulsivity and their relationships to developmental trajectories of externalizing behaviors from childhood into early adolescence

**DOI:** 10.1016/j.jrp.2024.104555

**Published:** 2024-12-10

**Authors:** M. Jia-Richards, A. Versace, R.L. Bachrach, F.L. Wang

**Affiliations:** a Department of Psychiatry, University of Pittsburgh, USA; b Diffusion Imaging Core of the Magnetic Resonance Research Center, University of Pittsburgh, USA; c Division of General Internal Medicine, University of Pittsburgh School of Medicine, USA; d Center for Health Equity Research and Promotion, Mental Illness Research, Education, and Clinical Center, VA Pittsburgh Healthcare System, USA

**Keywords:** Externalizing behaviors, Impulsivity, Children, Adolescents, Development

## Abstract

Impulsivity is a risk factor for externalizing behaviors, although the unique relationships between specific impulsivity facets and externalizing behavior development are less clear. We used Adolescent Brain Cognitive Development study data (*N = 11,874*) to examine whether child-reported UPPS-P impulsivity facets (9–10 years old) predicted parent-reported externalizing behaviors from childhood to early adolescence (11–12 years old). Latent growth model results showed that externalizing behaviors decreased over time. Higher negative urgency and lack of premeditation predicted greater externalizing behaviors in both childhood and early adolescence, as well as steeper declines in externalizing behaviors across time. Among the UPPS-P impulsivity facets, negative urgency and lack of premeditation may represent the most prominent indicators of externalizing behavior risk and development, highlighting their importance for targeted interventions.

## 1. Introduction

Adolescence is considered a sensitive period of development due to the multitude of biological, behavioral, emotional, and social developmental changes occurring during this time (Crone & Dahl, 2012; Fuhrmann et al., 2015; Larsen & Luna, 2018). Adolescence is also considered a period of heightened vulnerability for externalizing behaviors—e.g., aggression, delinquency, substance use, and other disruptive rule-breaking behaviors (Achenbach & Edelbrock, 1991; Achenbach et al., 2016)—as the ability to self-regulate behaviors and emotions is still maturing (Pas et al., 2021; Steinberg, 2010; Tarullo et al., 2009; Vazsonyi & Huang, 2010; Zondervan-Zwijnenburg et al., 2020). Better understanding childhood predictors of adolescent externalizing behaviors could help reduce the risk of more serious externalizing behaviors in adolescence that have long-lasting consequences, such as poorer academic, social, and mental health outcomes in adolescence and adulthood.

Disorders on the externalizing spectrum include attention deficit hyperactivity disorder (ADHD), oppositional defiant disorder (ODD), and conduct disorder (CD) (Alex Mason et al., 2010; Erskine et al., 2016; Henry et al., 2012). The symptoms underlying ADHD, ODD, CD, and other externalizing disorders are known to occur on a spectrum (Witkiewitz et al., 2013). Measuring externalizing behaviors as a dimensional construct rather than as discrete categories captures the substantial variability that exists in children and adolescents, while also allowing for more well-powered analyses of its risk factors. Therefore, for the purposes of the current study, we will focus our attention on levels of general externalizing behaviors to better understand the factors involved in externalizing behavior development.

### 1.1. Trait impulsivity facets

Trait impulsivity refers to a range of personality traits that reflect an individual’s tendency to engage in behaviors that are acted upon without forethought, with unnecessary risk, and with little regard for the potential consequences (DeYoung & Rueter, 2010; Evenden, 1999). Trait impulsivity is also an established and robust risk factor for high levels of externalizing behaviors across the lifespan (Beauchaine et al., 2017; Casey et al., 2008; Keil & Price, 2006; Krueger et al., 2009; Olson et al., 1999). Externalizing behaviors often develop concurrently and externalizing problems are often comorbid (Diamantopoulou et al., 2011), which has led some to theorize that impulsivity may be an underlying common latent risk factor (Beauchaine & McNulty, 2013; Beauchaine et al., 2017; Krueger et al., 2009).

Trait impulsivity is strongly associated with psychopathology across the spectrum of externalizing disorders, especially the emotionally driven aspects of impulsivity (Beauchaine et al., 2017; Berg et al., 2015; Carver & Johnson, 2018; Johnson et al., 2013). The Acquired Preparedness Model suggests that impulsivity shapes how people interact with their environment and learn from past experiences, which in turn may promote engaging in externalizing behaviors in the future (Doran et al., 2013; Halvorson et al., 2023; Settles et al., 2010). For example, someone high in sensation seeking may more likely be exposed to certain behaviors (e.g., substance use) due to seeking out novel experiences and developing positive expectancies from that experience and increasing the likelihood of continuing to engage in the behavior (Settles et al., 2010; Waddell et al., 2022). Therefore, it is important to study the longitudinal effects of trait impulsivity on externalizing behaviors.

One well-established conceptualization of trait impulsivity is the UPPS-P model of impulsivity (Lynam et al., 2006; Whiteside & Lynam, 2001). The UPPS-P model defines five unique facets: negative urgency, positive urgency, lack of premeditation, lack of perseveration, and sensation seeking. *Negative urgency* and *positive urgency* refer to the tendencies to act rashly in response to extreme negative and positive emotions, respectively. *Lack of premeditation* refers to the tendency to act without thinking or planning. *Lack of perseveration* refers to difficulties staying focused and completing tasks. Lastly, *sensation seeking* refers to the tendency to seek out novel and rewarding experiences. Each of these traits have, on their own, shown relationships with elevated externalizing problems in adolescent and adult populations.

Certain impulsivity facets may be more impactful than others in predicting externalizing problems in childhood and adolescence, which is important when considering the most effective strategies for prevention and intervention. Findings from cross-sectional studies using the UPPS-P framework have been somewhat mixed. Two studies found that self-reported higher negative urgency and lack of premeditation had the strongest relationships with self and teacher-reported externalizing behaviors in 9–14 year olds (Marmorstein, 2013; Watts et al., 2020). Another found that self-reported positive urgency and sensation seeking had the strongest relationships with self-reported externalizing behaviors in 14–18 year olds (Maneiro et al., 2017), however whether findings from 14 to 18-year-olds can be generalized to 9–10-year-olds is unclear.

Research using other measures capturing impulsivity and self-regulation may also be informative of how UPPS-P impulsivity dimensions predict externalizing problems, as UPPS-P traits have been shown to have empirical and content overlap with a wide range of these related measures (Sharma et al., 2014). For example, researchers have found that negative emotionality, which is similar to negative urgency, was associated with greater externalizing problems in across a range of developmental periods (Eisenberg et al., 2000, 2001, 2009; King et al., 2018; Lengua, 2002; Lengua et al., 1998; Lipscomb et al., 2012; Rabinowitz et al., 2016; Singh & Waldman, 2010; Wang et al., 2016). Many additional studies have also shown that effortful control and impulsivity (whose operationalizations were conceptually similar to lack of premeditation), were associated with elevated externalizing behaviors (Beauchaine et al., 2017; Creswell et al., 2019; Eisenberg et al., 2005, 2015; Lengua, 2002; Martel et al., 2017; Olson et al., 2005; Valiente et al., 2003). Although studies have shown an association between positive emotionality (e.g., sensation seeking, positive urgency) and externalizing problems (Cyders & Smith, 2007; Gilbert, 2012; Marmorstein, 2013; Smith & Cyders, 2016), research has also shown that the effect of positive emotionality attenuates once measures of negative emotion were added to the model (Kim et al., 2007). Taken together, negative urgency and lack of premeditation may lead to the expression of externalizing behaviors through poor regulation of negative emotions and a lack of consideration regarding the consequences of one’s actions.

### 1.2. Impulsivity and externalizing behaviors in childhood and adolescence

Changes in externalizing problems across development provide a more comprehensive picture of individuals’ overall functioning, and facets of impulsivity may be particularly important for predicting trajectories of externalizing problems. The most common finding in the literature suggests that average levels of parent-reported externalizing behaviors decline from childhood into adolescence (Bongers et al., 2004; Brieant et al., 2022a; Hatoum et al., 2018; Leve et al., 2005; Muratori et al., 2016; Rankin Williams et al., 2009; Van den Akker et al., 2013), although others have found that externalizing behaviors increase or stay relatively stable over time (Bos et al., 2018; Brieant et al., 2022b; Galambos et al., 2003; Keiley et al., 2001; López-Romero et al., 2015; Petersen et al., 2015; Yoon et al., 2021). Discrepancies between studies may be explained by use of different measures, or definitions of, externalizing behaviors, or by the age ranges studied.

In early childhood, aggressive externalizing behaviors like hitting and biting may be more common than in other developmental periods and decline as children learn how to use more effective coping strategies (Cole et al., 1996; Ersan, 2020). Children may also reduce their aggressive behaviors when they enter school to avoid peer rejection (Evans et al., 2019). In contrast, some research shows that rule-breaking externalizing problems may begin to rise as early as late childhood (Bongers et al., 2004), with higher levels in adolescence (Lahey et al., 2000). Status violations, (e.g., running away from home, truancy, substance use) appear to increase around 10 to 11 years-old, with these behaviors escalating throughout adolescence (Bongers et al., 2004). The commonly used externalizing scale from the parent-reported Child Behavior Checklist (CBCL; (Achenbach & Edelbrock, 1991) measures both rule breaking (e.g., status violations) and aggressive behaviors (e.g., bullying). As rule breaking behaviors begin to rise in adolescence, parent-reported CBCL externalizing scores could reflect decreasing aggression from childhood to early adolescence—the focus of the current study—and thus may be more likely to measure declines over time.

Similar to prior work examining prediction of levels of externalizing behaviors, studies have shown that measures conceptually similar to negative urgency and lack of premeditation might pose greatest risk for *growth* in externalizing problems. For instance, Perry et al (2018) found that behavioral inhibitory control (conceptually similar to lack of premeditation) and emotion regulation (with parallels to negative urgency) were important in distinguishing between children who showed normative declines vs. consistently high levels from early childhood to adolescence. In a younger sample, lower levels of effortful control (i.e., lack of premeditation, perseverance) were associated with smaller declines in externalizing behaviors from 3 to 9 years old (Nielsen et al., 2019). However, we note that this literature is mixed, as others have found that poorer self-regulation was associated with *greater* declines in externalizing behaviors from adolescence to young adulthood (Chen & Vazsonyi, 2011), that there was a role for positive emotionality (i.e., sensation seeking, positive urgency) in predicting declines in externalizing problems (Nielsen et al., 2019), and that effortful control and impulsivity (i.e., lack of premeditation) did not predict trajectories of externalizing problems from childhood to adolescence (King et al., 2013). Although research points towards possible roles of negative urgency and lack of premeditation, more work is needed examining the joint effects of all five UPPS-P facets in predicting growth in externalizing problems from childhood to adolescence.

### 1.3. Study Objective

The current study aimed to better understand the effect of different facets of impulsivity in childhood on trajectories of parent-reported externalizing behaviors from childhood (9 – 10 years old) into early adolescence (11 – 12 years old) using available data from the Adolescent Behavior Cognitive Development (ABCD) study (Casey et al., 2018)—a longitudinal study of biological and behavioral development and the impact of environmental, biological, and social factors on adolescent development and health in a sample of 11,876 youth (Volkow et al., 2018). Based on previous findings from ABCD study data, we expected externalizing behaviors to decline over time (Brieant et al., 2022a), and that negative urgency and lack of premeditation would be uniquely and positively associated with externalizing behaviors cross-sectionally at 9–10 years old (Watts et al., 2020). We further hypothesized that negative urgency and lack of premeditation would be among the facets significantly associated with smaller maturational declines in externalizing behaviors from 9–10 years old to 11–12 years old, although it is unclear from the current literature whether these two facets would be the only ones predicting smaller declines in externalizing behavior. For example, positive urgency may also play a role (Nielsen et al., 2019; Perry et al., 2018).

## 2. Method

This study was not preregistered.

### 2.1. Sample

Participants were drawn from the ongoing longitudinal ABCD study. Beginning in 2016, a total of 11,876 demographically diverse youth aged 9 to 10 years old were recruited from 21 sites across the United States (Garavan et al., 2018). We used data from the 4.0 data release collected at baseline (T1; 9–10 years old), one-year (T2; 10–11 years old), and two-year (T3; 11–12 years old) follow-up assessments, including participants’ trait impulsivity data at baseline (T1) and externalizing behavior data at each time point (T1–T3, see *Measures*). Two participants were removed from analyses as they had missing externalizing data for all time points, resulting in a final sample size of 11,874 for the current study. Of the final sample, 48 % were female sex assigned at birth, 52 % White, 15 % Black, 20 % Hispanic, 2 % Asian, and 11 % reported “other” or multiple racial and ethnic backgrounds (see Table 1 for full sociodemographic characteristics).

### 2.2. Materials

#### 2.2.1. Participant characteristics

Participant characteristics were reported at T1, or at T2 if data were missing at T1, and were included as covariates in analyses.

##### Background and Demographics.

Parents reported participants’ sex assigned at birth (Potter et al., 2022), racial and ethnic identity (Asian, Black, White, Hispanic, and “Other” which includes more than one race and ethnicity), household income (<$50,000, $50,000 – $100,000, >$100,000), and parental education level (<bachelor’s degree, bachelor’s degree, graduate degree) (Barch et al., 2018).

##### Neighborhood Disadvantage.

The Area Disadvantage Index (ADI) is derived from U.S. Census data on income, housing, education, and employment by zip code (Singh, 2003). Scores were scaled with a mean of 100 and SD of 20, with higher scores indicating greater neighborhood disadvantage (Kind et al., 2014).

##### Pubertal Development.

Mean scores on the parent-rated Pubertal Development Scale (PDS) (Petersen et al., 1988) were used to measure participants’ pubertal development, with higher scores indicating more advanced pubertal status.

#### 2.2.2. Externalizing behaviors

The parent-rated externalizing scale from the Child Behavior Checklist (CBCL) (Achenbach & Edelbrock, 1991) was used to measure externalizing behaviors annually at T1, T2, and T3. The CBCL has a total of 112 items that are used to derive several scales, including externalizing behaviors. Parent(s) rate items describing common problem behaviors in youth on a scale from 0 (not true) to 2 (very or often true). Item ratings are summed to calculate raw scores and *T* scores, with higher numbers indicating higher levels of externalizing behaviors. Raw scores were used in analyses for the current study. Internal consistency for the full CBCL in the ABCD sample was excellent (*α* = 0.95 for T1, T2, and T3).

#### 2.2.3. Trait impulsivity

The 20-item abbreviated youth UPPS-P impulsivity scale (Lynam et al., 2006; Watts et al., 2020; Whiteside & Lynam, 2001) was used to measure negative urgency, positive urgency, lack of premeditation, lack of perseveration, and sensation seeking at T1. The UPPS-P was also administered at T3, which we used in post-hoc analyses. Youth rated items on a scale from 1 (disagree strongly) to 4 (agree strongly). Items were then summed to produce scores for each facet. The abbreviated youth UPPS-P has demonstrated similar psychometric properties as the original version in the ABCD sample (Watts et al., 2020). Internal consistency (α) for each facet ranged from poor to good: negative urgency (0.63), positive urgency (0.78), lack of premeditation (0.73), lack of perseveration (0.70), and sensation seeking (0.50).

#### 2.2.4. Analyses

Latent growth models were fit in R ver. 4.4.0 (R Core Team, 2024) with *lavaan* (Rosseel, 2012) and full estimation maximum likelihood (FIML) estimation. Chi-square, the root mean square error of approximation (RMSEA), the comparative fit index (CFI), and the Tucker-Lewis index (TLI) were used to assess model fit (Iacobucci, 2010; Schermelleh-Engel et al., 2003; Shi & Maydeu-Olivares, 2020).

We first estimated the average linear trajectory (latent intercept and slope) of externalizing behaviors from T1 to T3 (Model 1, the unconditional model). We then examined whether the UPPS-P impulsivity facets were associated with the intercept and/or slope of the estimated average linear trajectory (Model 2, the conditional model). More advanced pubertal development (Dimler & Natsuaki, 2015; Steinberg et al., 2008; Ullsperger & Nikolas, 2017), male sex assigned at birth (Bongers et al., 2004; Cross et al., 2011; Fernandez Castelao & Kröner-Herwig, 2014; Weafer & de Wit, 2014), greater neighborhood disadvantage (Li et al., 2017; Lynam et al., 2000; Vazsonyi et al., 2006), lower parental education (Anderson et al., 2022; Auger et al., 2010), and financial insecurity (Anderson et al., 2022; Lynam et al., 2000) have been associated with higher levels of both externalizing problems and impulsivity in children and adolescents, potentially creating spurious associations between externalizing and impulsivity if not controlled for; therefore, these variables were included as covariates. Race and ethnicity were also included as a covariates to account for additional sociocultural factors that may not be captured by the other covariates, like experiencing racism (Loyd et al., 2019). See Table S3 for correlations between all covariates.

##### Handling of Missing Data.

In the ABCD sample, 58 % of participants had complete data for our variables included in analyses. Less than 1 % of participants had missing CBCL externalizing data at T1, 6 % had missing data at T2, and 31 % had missing data at T3. Less than 1 % of participants had missing UPPS-P data at baseline. Among the sociodemographic variables, 13 % had missing household income data, 7 % had missing neighborhood disadvantage data, and 6 % had missing parental education data. Missing data were associated with more advanced pubertal development (*r* = 0.08, *p* < 0.001), lower income (*r* = −0.07, *p* < 0.001), lower parental education (*r* = −0.10, *p* < 0.001), greater neighborhood disadvantage (*r* = 0.06, *p* < 0.001), higher negative and positive urgency (*r* = 0.03, *p* < 0.001; *r* = 0.05, *p* < 0.001), higher lack of perseveration (*r* = 0.03, *p* < 0.001), and higher levels of externalizing behaviors at T1 and T2 (*r* = 0.05, *p* < 0.001; *r* = 0.04, *p* < 0.001). Given these observed variables were associated with missingness, missing data were considered missing at random. Compared to White participants, participants of Black, Hispanic, and Other racial backgrounds had higher proportions of missing data. Compared to Hispanic, Asian, and Other racial backgrounds, Black participants had higher proportions of missing data (see Supplementary Table S1 for post-hoc contrasts). We ran latent growth models using complete data and different degrees of missing data with Full Information Maximum Likelihood (FIML) estimation and found that the differences between findings were negligible, even when including all the missing data (see Supplementary Table S2 for results using with no missingness), therefore the entire sample with missing data (using FIML) were retained in analyses and are presented below.

## 3. Results

### 3.1. Descriptive Statistics

Means and standard deviations for CBCL externalizing scores and the UPPS-P facets can be found in Table 1. Correlations between all study measures can be found in Supplementary Table S3.

### 3.2. Latent growth models

#### 3.2.1. Model 1 (Unconditional Model)

Table 2 contains the full results for the unconditional model. The model fit well—χ^2^(1) = 0.98, *p* = 0.322; CFI/TLI = 1.00/1.00; RMSEA = 0.000, 90 % CI[.000, 0.024], with fit indices passing established thresholds (Iacobucci, 2010; Shi & Maydeu-Olivares, 2020).

Mean levels of externalizing behaviors decreased linearly over time (unstandardized intercept = 4.44, *SE* = 0.05, 95 % CI [4.34, 4.55]; slope = −0.21, *SE* = 0.02, 95 % CI [−0.25, 0.16]). Variances for both the intercept and the slope were significant, indicating that there was significant heterogeneity in youths’ baseline (T1) levels of externalizing behaviors and the degree of change between T1 and T3. The intercept and slope significantly covaried such that higher levels of externalizing behaviors at T1 were associated with greater decreases in externalizing behaviors over time (*r* = −0.39, *p* < 0.001, 95 % CI [−0.40,−0.37]).

#### 3.2.2. Model 2 (Conditional Model): Intercept effects

**Fig. 1.**
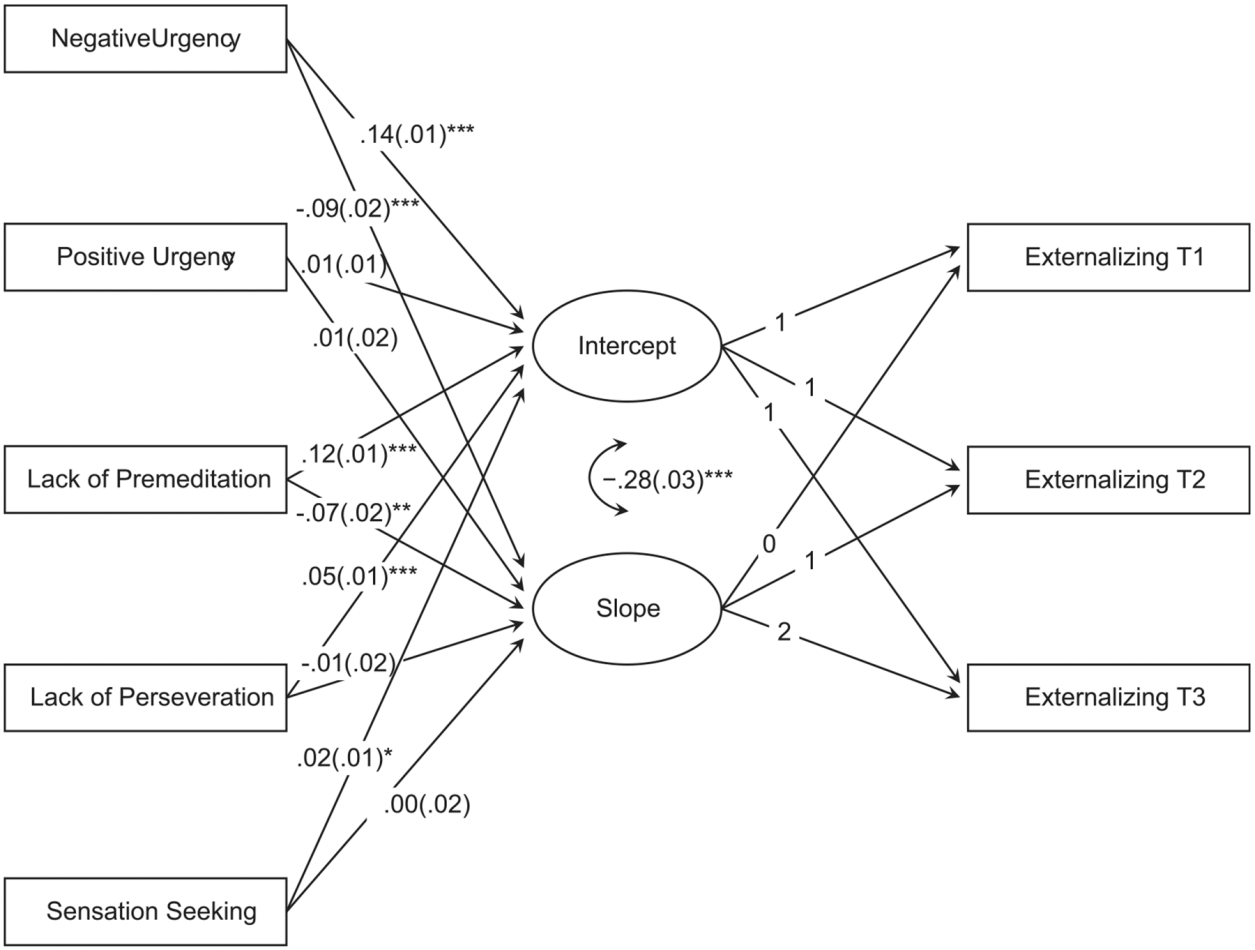
Results of the conditional model.

Table 3 contains the full results for the conditional model and Fig. 1 visualizes the findings. Model 2 fit well: χ^2^(15) = 16.53, *p* = 0.348; CFI/TLI = 1.00/1.00; RMSEA = 0.003, 90 % CI [.00, 0.01]. Of the UPPS-P facets, higher negative urgency, lack of premeditation, lack of perseveration, and sensation seeking were associated with higher levels of externalizing behaviors at T1. Of the sociodemographic covariates, more advanced pubertal status and living in a more disadvantaged neighborhood were associated with higher levels of externalizing behaviors at T1, while being female, higher household income, and higher parental education were associated with lower levels of externalizing behaviors at T1. In comparison to identifying as White, racially/ethnically identifying as Black, Asian, Hispanic, or Other was associated with lower levels of externalizing behaviors at T1. Altogether, the sociodemographic factors accounted for 4.6 % of variance in the intercept and the UPPS-P facets accounted for 5.1 % of variance. Negative urgency, lack of premeditation, and lack of perseveration accounted for 1.4 %, 1.1 %, and 1.7 % of the variance in the intercept, respectively.

#### 3.2.3. Model 2 (Conditional Model): Slope effects

**Fig. 2.**
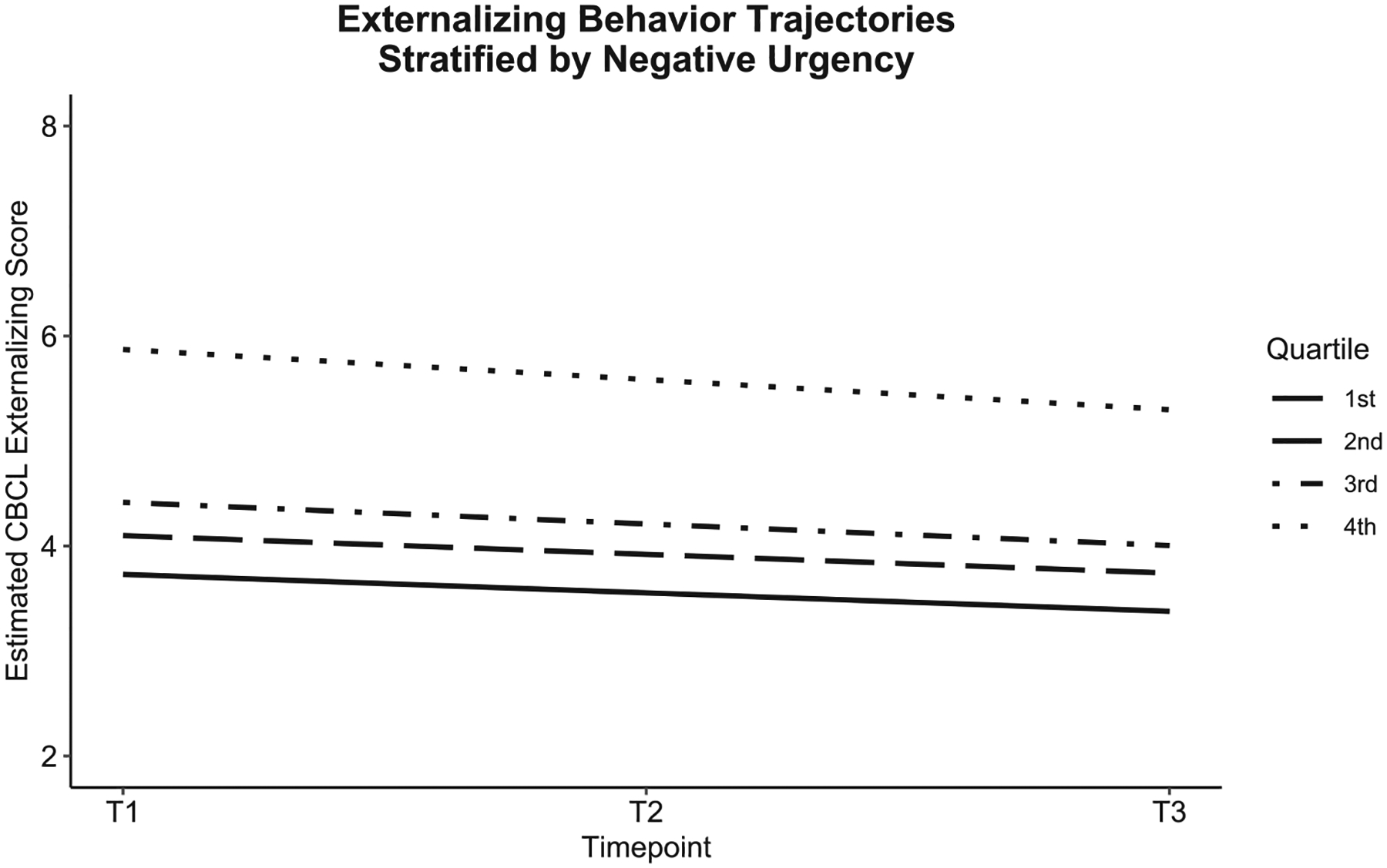
Externalizing behavior trajectories by levels of negative urgency.

**Fig. 3.**
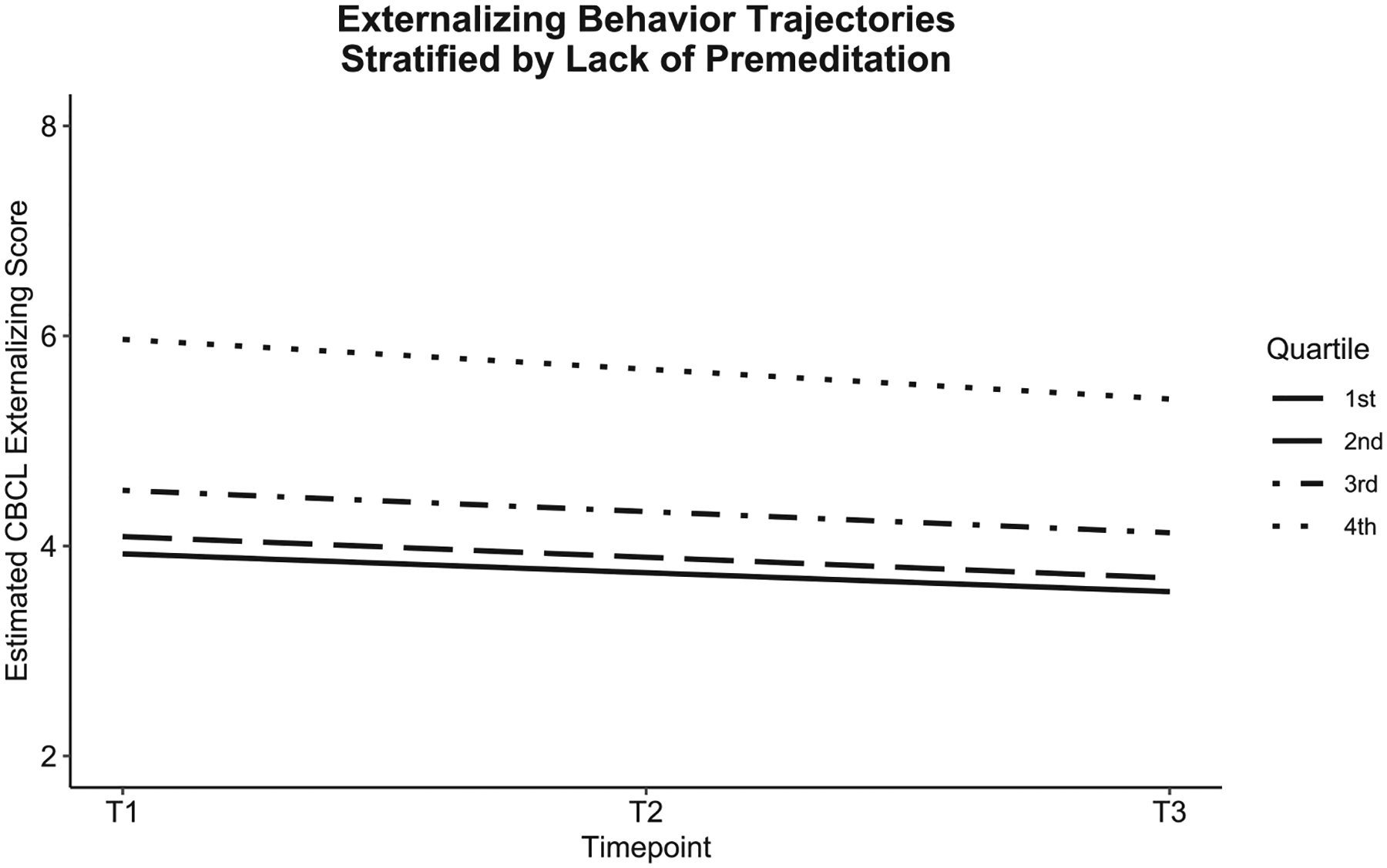
Externalizing behavior trajectories by levels of lack of premeditation.

Of the UPPS-P facets, higher negative urgency and higher lack of premeditation predicted more negative slopes. Higher levels of these facets were associated with greater declines in externalizing behaviors over time. Higher parental education was associated with smaller declines in externalizing behaviors, and identifying as Black was associated with greater declines over time (see Table 3). Altogether, the sociodemographic factors accounted for 2.0 % of variance in the slope. The UPPS-P facets accounted for 1.4 % of variance in the slope, with negative urgency and lack of premeditation accounting for 0.1% and 0.3% of the variance, respectively. Figs. 2 and 3 visualize the effects of negative urgency and lack of premeditation by plotting estimated externalizing trajectories, stratified by quartiles of each factor.

### 3.3. Post-Hoc analyses

#### 3.3.1. Sensitivity power analyses

Sensitivity power analysis was conducted to determine the minimum effect that could have been detected in our conditional models given the number of parameters being estimated (k = 36) when α = 0.05 and power = 0.80. The minimum effect was small, with *R*^2^ = 0.002.

#### 3.3.2. Suppression effects

Although negative and positive urgency represent two distinct facets (Cyders & Smith, 2008), they are still closely related constructs as they both represent the tendency for mood-based rash action (Billieux et al., 2021). They were also strongly correlated in our sample (*r* = 0.49, *p* < 0.001). Therefore, to determine whether our findings were affected by multicollinearity, we ran two conditional models removing either negative or positive urgency as a covariate. Results from these models can be found in supplementary tables S4 and S5. Both models fit equally well. Removing positive urgency from the model left the remaining effects effectively unchanged. When removing negative urgency from the model, the effect of positive urgency at the slope changed from a positive to negative effect, indicating a suppression effect and suggesting that positive urgency may have little effect on externalizing behaviors in our sample. The effect remained non-significant, and the remainder of the model was effectively unchanged. The effect of positive urgency at the slope should be interpreted with caution, however, the main findings in Model 2 remain unchanged.

#### 3.3.3. Negative urgency and lack of premeditation predicting externalizing behaviors at T3

Based on the finding that higher levels of negative urgency and lack of premeditation predicted greater declines in externalizing behaviors over time, we wanted to examine whether youth high in these facets continued to show elevated levels of externalizing behaviors at T3. We respecified Model 2, placing the intercept at T3 (an equivalent model to Model 2) and found that negative urgency (standardized est. = 0.09, *SE* = 0.03, *p* < 0.001, 95 % CI [.04, 0.14]) and lack of premeditation (std. est. = 0.07, *SE* = 0.02, *p* = 0.006, 95 % CI [.02, 0.11]) still predicted greater externalizing behaviors at T3. In other words, although youth with higher negative urgency and lack of premeditation had the greatest declines in externalizing behaviors over time, they still had higher mean levels of externalizing behaviors in adolescence compared to youth lower in these UPPS-P facets.

#### 3.3.4. Bivariate latent change score models

We also sought to understand whether changes in negative urgency and lack of premeditation were associated with changes in externalizing behaviors. If so, this could suggest that the maturational processes underlying externalizing behavior development may similarly underlie developmental changes in negative urgency and lack of premeditation and provide a possible explanation for our findings. In the ABCD study, the UPPS-P is administered every other year (i.e., T1 and T3), therefore we conducted two bivariate latent change score models using data from T1 and T3 to see whether changes in negative urgency (mean score at T1 = 8.49, *SD* = 2.34; mean score at T3 = 7.77, *SD* = 2.34) and lack of premeditation (mean score at T1 = 7.74, *SD* = 2.38; mean score at T3 = 7.77, *SD* = 2.24) were associated with changes in externalizing behaviors between T1 and T3. Models also controlled for sociodemographic covariates. The remaining UPPS-P facets were not included as the resulting model fits were poor. Fit indices for the negative urgency model were χ^2^(16) = 675.15, *p* < 0.000; CFI/TLI = 0.91/.80; RMSEA = 0.059, 90 % CI[.055, 0.065], and fit indices for the lack of premeditation model were χ^2^(16) = 818.21, *p* < 0.000; CFI/TLI = 0.91/.78; RMSEA = 0.065, 90 % CI[.061, 0.069].

Estimated mean levels of negative urgency (standardized Δest. = 2.40, *SE* = 0.07, *p* < 0.001, 95 % CI [2.26, 2.54]) and lack of premeditation (standardized Δest. = 2.20, *SE* = 0.07, *p* < 0.001, 95 % CI [2.06, 2.33]) decreased significantly between T1 and T3 (Δest. refers to the degree of change between timepoints). Furthermore, estimated declines in both negative urgency (std. est. = 0.12, *SE* = 0.01, p < 0.001, 95 % CI [.10, 0.12]) and lack of premeditation (std. est. = 0.11, *SE* = 0.01, p < 0.001, 95 % CI [.09, 0.13]) were associated with declines in externalizing behaviors between T1 and T3.

## 4. Discussion

In a large and demographically diverse sample of youth, we found that, on average, externalizing behaviors in the ABCD sample decreased from 9–10 years old to 11–12 years; however, the significant variability of the estimated slopes indicates that some youth saw increases in their externalizing behaviors. Youth with the highest levels of externalizing behaviors in childhood showed the greatest declines in externalizing over time. We also found that the five UPPS-P facets of impulsivity were differentially related to developmental trajectories of externalizing behaviors across childhood and early adolescence, with negative urgency and lack of premeditation being the only significant predictors of changes in externalizing behaviors over time, controlling for all five UPPS-P facets.

Decreases in average levels of externalizing problems are consistent with prior research (Bongers et al., 2004; Brieant et al., 2022a; Hatoum et al., 2018; Leve et al., 2005; Muratori et al., 2016; Rankin Williams et al., 2009; Van den Akker et al., 2013). Given the age range of the sample, aggressive behaviors might be the driving force behind the decreases in externalizing behaviors. There was a significant degree of variability in youth’s baseline levels of externalizing behaviors and their trajectories, suggesting that there may be multiple subclasses of trajectories (e.g., increasing, decreasing, or stable) with varying levels of childhood externalizing behaviors within the sample. Of the UPPS-P facets, only negative urgency and lack of premeditation predicted externalizing behavior trajectories, underscoring the importance of these two impulsivity facets in the development of externalizing behaviors. Negative urgency and lack of premeditation predicted greater declines, or smaller increases, in externalizing behaviors over time, which may be explained by their larger effect sizes on the intercept (negative urgency Δ*R*^2^ = 0.014, lack of premeditation Δ*R*^2^ = 0.011) compared to other facets (Δ*R*^2^ range: <.001 – 0.002), and the significant negative covariance between intercepts and slopes of trajectories. Childhood negative urgency and lack of premeditation also predicted higher levels of externalizing behaviors across timepoints, suggesting that they are the two strongest impulsivity markers for youth at high risk for externalizing problems, out of the UPPS-P facets. Our post-hoc analyses showed that developmental changes in these two impulsivity facets appear to track with developmental changes in externalizing behaviors from 9 to 10 to 11 – 12 years old. Externalizing behaviors, negative urgency, and lack of premeditation could be indicators of the same underlying developmental processes as youth mature, with negative urgency and lack of premeditation serving as targets for intervention or prevention efforts.

Our findings indicate that impulsive reactivity to negative emotions and a tendency to act without forethought in childhood are predictive of externalizing behaviors into adolescence, corroborating prior studies that have demonstrated similar relationships with measures of emotion regulation and behavioral inhibition (Perry et al., 2018). Our findings also echo a review by Modecki et al. (2017) concluding that emotion regulation, coping, and decision-making are linked to externalizing behaviors in adolescents, and that intervening on these abilities could effectively reduce the risk of externalizing problems. Cross-sectional work in college students has similarly demonstrated that lack of follow-through and behavioral reactivity to emotions are related to externalizing-related psychopathology (e.g., aggression and substance use; Johnson et al., 2017). These studies, however, did not distinguish between negative and positive emotional reactivity. Negative and positive urgency are closely related by virtue of being indicators of emotional reactivity, and both are related to a broad range of psychopathologies (Berg et al., 2015), which has led some to question the utility of measuring them as two separate constructs (Billieux et al., 2021). Results from the current study highlight the importance of reactivity to negative emotions to externalizing behaviors in the childhood to adolescence developmental period. Reactivity to positive emotions may instead be more closely tied to gambling behaviors (Willie et al., 2022), which are not represented in the CBCL’s externalizing scale, or to manic symptoms in bipolar disorders (Giovanelli et al., 2013).

Negative urgency and lack of premeditation could be particularly important targets for intervention and prevention and addressing them may need to be prioritized above other facets of impulsivity. Although the proportion of variance explained in externalizing trajectories by negative urgency (Δ*R*^2^ = 0.1) and lack of premeditation (Δ*R*^2^ = 0.4%) in our study was small, the effects found in large and diverse samples like ABCD tend to be smaller than the effect sizes one would expect to find in smaller more homogenous samples using traditional effect size metrics (Owens et al., 2021), and even small effects can create meaningful changes over time (Funder & Ozer, 2019). However, the small effects we found may only be significant given our relatively large sample size, which leaves uncertainty about their clinical significance. Additionally, although emotion regulation is associated with externalizing psychopathology, we found that this relationship may be specific to regulating negative emotions in childhood and adolescence. Teaching children strategies to cope with negative emotions and improve decision making capabilities could help reduce externalizing behaviors into adolescence, prior to the onset of more consequential behaviors.

## 5. Strengths and Limitations

A major strength of the current study was the use of longitudinal data. Although cross sectional studies have previously demonstrated similar associations between the UPPS-P facets and externalizing behaviors, longitudinal findings help establish early predictors of change and can inform prevention and interventions for youth most in-need. Another major strength was the use of data from the ABCD sample, whose recruitment procedures aimed to capture the natural variation and diversity within the U.S. population (Garavan et al., 2018), resulting in a large sample of demographically diverse youth, and increasing the generalizability of our findings. Additionally, the combination of child-reported trait impulsivity and parent-reported externalizing behaviors lessens the likelihood of reporter bias and helps demonstrate the validity of children rating their own impulsive traits.

There are additional unmeasured factors that could explain our findings. For instance, youth with high levels of externalizing behaviors and/or impulsivity may have been more likely to receive pharmacological or psychological treatment to reduce problems related to externalizing behaviors and impulsivity. Given that the relationship between parenting style and youth behavior is reciprocal, changes in parenting style or involvement as youth mature may also affect development (Kerr et al., 2012). Transitioning to a new school for middle school also opens opportunities to form new peer groups which could influence behavioral development. For example, youth who transition to a new school report fewer instances of bullying (Farmer et al., 2011). Additionally, longitudinal data were unavailable for parental education and household income, both of which could change over time and affect our findings.

Our findings are limited in terms of how externalizing behaviors were measured during the child and adolescent developmental periods captured in the study. Externalizing behaviors were parent-reported using the CBCL, which is a commonly used measure of behavioral problems in youth. On the CBCL, adolescents tend to report higher levels of externalizing behaviors compared to parent report (Sourander et al., 1999); thus, parent-report may underestimate the severity of externalizing behaviors in adolescence. Parents may also be less aware of certain behaviors that tend to emerge in adolescence. For example, while studies have found that around 50 % of parents are aware of their adolescent child using substances, parents tend to be less aware of the frequency of substance use and any substance use-related problems (Fisher et al., 2006; McGillicuddy et al., 2007). Factors like an insecure parental attachment may also affect parental knowledge of their adolescent’s behavior (Jones et al., 2015).

Another limitation of the study was the degree of missing data, particularly at the later time points. Youth who had missing data typically had high levels of externalizing behaviors and more disadvantaged socioeconomic backgrounds. These youth may be among the highest risk for elevated externalizing behaviors across childhood and adolescence, however they are also more likely to miss assessments as the study progresses (Ewing et al., 2022). Their missing data at later timepoints may have biased our findings towards lower risk youth. However, we tried to counteract this bias by avoiding listwise deletion as much as possible and using FIML to estimate the missing data.

While also a strength of the study, using a child-reported measure of trait impulsivity may have limited our findings as the internal consistencies for some of the facets on the UPPS-P scale were low. In particular, the sensation seeking facet had poor internal consistency, which could explain our lack of findings for this facet. Finally, although externalizing behaviors may follow non-linear trends (Bongers et al., 2004; Petersen et al., 2015; Reef et al., 2011), our analyses were restricted to linear growth trajectories as we only had data across three timepoints.

## 6. Conclusions

Our study is one of a few to examine the longitudinal relationships between facets of impulsivity in childhood and externalizing behavior trajectories into early adolescence using a large and demographically diverse sample of youth in the United States. Negative urgency and lack of premeditation may be the most exigent aspects of impulsivity to target to effectively alter the trajectory of externalizing behaviors during this sensitive period of development. Moreover, targeting these facets in childhood, when the ability to regulate emotions and behaviors is still developing, may have longer lasting effects on behavior over time.

## Supplementary Material

Supplementary Material

## Figures and Tables

**Table 1:** Percentages, means, and standard deviations for participant characteristics, sociodemographic variables, and study measures (N = 11,874).

Sample Characteristics	%, range, or *M* (*SD*)	Predictors and Outcomes	*M* (*SD*)
Sex		UPPS-P Facets (T1)	
Female	48 %	Negative Urgency	8.49 (2.65)
Male	52 %	Positive Urgency	7.99 (2.96)
Race/Ethnicity		Lack of Premeditation	7.74 (2.38)
White	52 %	Lack of Perseveration	7.04 (2.25)
Black	15 %	Sensation Seeking	9.77 (2.68)
Hispanic	20 %	CBCL Externalizing (Raw)	
Asian	2 %	T1 (9–10 years)	4.45 (5.86)
Multiple or Other	11 %	T2 (10–11 years)	4.18 (5.66)
Mean PDS	1.61 (0.50)	T3 (11–12 years)	3.93 (5.52)
Median Household Income	$75,000–$99,000	CBCL Externalizing (*T* Score)	
Minimum	<$5,000	T1 (9–10 years)	45.72 (10.33)
Maximum	>$200,000	T2 (10–11 years)	45.21 (10.13)
Parent Education		T3 (11–12 years)	44.48 (9.80)
< High School Degree	6 %		
High School Degree	24 %		
Associate’s degree	13 %		
Bachelor’s Degree	27 %		
Graduate Degree	25 %		
Neighborhood (ADI)	94.64 (21.15)		

*Note*. PDS = Pubertal Development Scale; ADI = Area Disadvantage Index; UPPS-P = Abbreviated Youth UPPS-P Impulsivity Scale; CBCL = Child Behavior Checklist.

**Table 2:** Unstandardized and standardized parameter estimates for Model 1 (unconditional model).

Parameter	Est. (*SE*)	95 % CI	Std. Est (*SE*)	95 % CI	*p*
**Means**					
Intercept (T1)	4.44 (0.05)	4.34, 4.55	0.86 (0.01)	0.83, 0.88	0<.001
Slope	−0.21 (0.02)	−0.25, −0.16	−0.19 (0.03)	−0.24, −0.14	0<.001
**Variances**					
Intercept (T1)	26.84 (0.53)	25.81, 27.88	1.00 (0.00)	1.00, 1.00	0<.001
Slope	1.26 (0.21)	0.86, 1.67	1.00 (0.00)	1.00, 1.00	0<.001
Externalizing (T1)	7.54 (0.41)	6.74, 8.35	0.22 (0.01)	0.20, 0.24	0<.001
Externalizing (T2)	7.96 (0.21)	7.55, 8.37	0.25 (0.01)	0.24, 0.26	0<.001
Externalizing (T3)	7.13 (0.41)	6.32, 7.93	0.23 (0.01)	0.20, 0.25	0<.001
**Covariance**					
Intercept/Slope	−1.86 (0.26)	−2.37, −1.34	−0.32 (0.02)	−0.37, −0.27	0<.001

**Table 3:** Standardized parameter estimates for Model 2 (conditional model).

Parameter	Std. Est. (SE)	*p*	95 % CI	Δ*R*^*2*^
**Intercept Effects (T1)**				
Sociodemographic Characteristics				0.056
Sex	−0.11 (0.01)	0<.001	−0.13, −0.09	0.010
PDS	0.03 (0.01)	0.003	0.01, 0.05	0.008
Income	−0.18 (0.01)	0<.001	−0.21, −0.15	0.016
Parent Education	−0.04 (0.01)	0.001	−0.07, −0.02	0.001
ADI	0.02 (0.01)	0.037	0.00, 0.05	0.014
Race/Ethnicity				0.005
Black	−0.04 (0.01)	0.002	−0.06, −0.01	0.000
Asian	−0.06 (0.01)	0<.001	−0.08, −0.04	0.002
Hispanic	−0.04 (0.01)	0<.001	−0.06, −0.02	0.003
Other	0.02 (0.01)	0.044	0.00, 0.04	0.000
UPPS-P				0.051
Negative Urgency	0.14 (0.01)	0<.001	0.12, 0.16	0.014
Positive Urgency	0.01 (0.01)	0.381	−0.01, 0.03	0.000
Lack of Premeditation	0.12 (0.01)	0<.001	0.10, 0.14	0.011
Lack of Perseveration	0.05 (0.01)	0<.001	0.03, 0.07	0.002
Sensation Seeking	0.02 (0.01)	0.031	0.00, 0.04	0.000
**Slope Effects (T1 – T3)**				
Sociodemographic Characteristics				0.020
Sex	0.04 (0.02)	0.060	0.00, 0.09	0.002
PDS	0.02 (0.02)	0.329	−0.02, 0.07	0.001
Income	0.05 (0.03)	0.087	−0.01, 0.12	0.001
Parent Education	0.07 (0.03)	0.015	0.01, 0.12	0.003
ADI	0.02 (0.02)	0.487	−0.03, 0.07	0.000
Race/Ethnicity				0.006
Black	−0.06 (0.03)	0.022	−0.11, −0.01	0.003
Asian	0.03 (0.02)	0.214	−0.02, 0.08	0.001
Hispanic	0.02 (0.02)	0.294	−0.02, 0.06	0.001
Other	0.00 (0.02)	0.869	−0.04, 0.05	0.000
UPPS-P				0.014
Negative Urgency	−0.09 (0.02)	0<.001	−0.14, −0.04	0.001
Positive Urgency	0.01 (0.02)	0.807	−0.04, 0.05	0.000
Lack of Premeditation	−0.07 (0.02)	0.006	−0.11, −0.02	0.003
Lack of Perseveration	−0.01 (0.02)	0.734	−0.05, 0.04	0.000
Sensation Seeking	0.00 (0.02)	0.958	−0.04, 0.04	0.003

*Note*. Δ*R*^*2*^ was calculated by comparing the *R*^*2*^ of models with and without the covariate as an estimate of effect size. Reference groups for categorical variables: Sex = Male, race/ethnicity = White. PDS = Pubertal Development Scale; ADI = Area Disadvantage Index; UPPS-P = Abbreviated Youth UPPS-P Impulsivity Scale.

## Data Availability

Data used in this article were obtained from the Adolescent Brain Cognitive Development (ABCD) Study, held in the National Institutes of Mental Health Data Archive (https://nda.nih.gov/).

## Appendix A. Supplementary material

Supplementary data to this article can be found online at https://doi.org/10.1016/j.jrp.2024.104555.
