# Supplementary material for: Facets of trait impulsivity and their relationships to developmental trajectories of externalizing behaviors from childhood into early adolescence
Source: J Res Pers. Author manuscript; Available in PMC 2026 May 1. (PMC13132145; doi:10.1016/j.jrp.2024.104555)
Supplement: Supplementary Material [file NIHMS2168358-supplement-Supplementary_Material.docx]

**Supplementary Materials**

Supplementary materials for: Jia-Richards, M. et al. Facets of trait impulsivity and their relationships to developmental trajectories of externalizing behaviors from childhood into adolescence.

**Table S1**

*Post-Hoc Contrasts for Comparing the Proportion of Missing Data Between Racial Groups*

| **Contrast** | **Cohen’s *d*** | ***SE*** | ***t*(11,870)** | ***p_adj_*** |
| --- | --- | --- | --- | --- |
| White - Black | -.030 | 0.002 | -19.89 | <.0001 |
| White - Hispanic | -0.015 | 0.001 | -11.29 | <.0001 |
| White - Asian | -0.005 | 0.004 | -1.28 | 0.701 |
| White - Other | -0.011 | 0.002 | -6.17 | <.0001 |
| Black - Hispanic | 0.015 | 0.002 | 8.43 | <.0001 |
| Black - Asian | 0.025 | 0.004 | 6.72 | <.0001 |
| Black - Other | 0.019 | 0.002 | 9.30 | <.0001 |
| Hispanic - Asian | 0.011 | 0.004 | 2.85 | 0.036 |
| Hispanic - Other | 0.004 | 0.002 | 2.29 | 0.149 |
| Asian - Other | -0.006 | 0.004 | -1.58 | 0.512 |
| *Note.* Tukey-adjusted *p*-values. | | | | |

**Table S2**

*Estimates of Model Parameters in Participants with Complete Data (N = 6,923).*

| Unconditional Model Fit | | | | | | | |
| --- | --- | --- | --- | --- | --- | --- | --- |
| χ2(1), *p* | CFI | | TLI | | RMSEA | | 90% CI |
| 2.22, *p* = .137 | 1.00 | | 1.00 | | .013 | | .000, .038 |
|  | | | | | | | |
| Unconditional Model Parameter Estimates | | | | | | | |
| Parameter | | Est. | | *SE* | | *p* | |
| Means | |  | |  | |  | |
| Intercept (T1) | | 4.29 | | .07 | | < .001 | |
| Slope | | -0.19 | | .03 | | .005 | |
| Variances | |  | |  | |  | |
| Intercept (T1) | | 22.85 | | .47 | | < .001 | |
| Slope | | 0.69 | | .24 | | < .001 | |
| T1 Externalizing | | 8.48 | | .45 | | < .001 | |
| T2 Externalizing | | 7.12 | | .60 | | < .001 | |
| T3 Externalizing | | 7.83 | | .23 | | < .001 | |
| Covariance | |  | |  | |  | |
| Intercept/Slope | | -1.07 | | 0.27 | | < .001 | |
|  | |  | | | | | |
| Conditional Model Fit | | | | | | | |
| χ2(45) | CFI | | TLI | | RMSEA | | 90% CI |
| 12558.87 | 1.00 | | .999 | | .006 | | .000, .014 |
|  | | | | | | | |
| Conditional Model Parameter Estimates | | | | | | | |
| Parameter | | Est. | | *SE* | | *p* | |
| Intercept Effects (T1) | |  | |  | |  | |
| Sociodemographics | |  | |  | |  | |
| Sex (Female) | | -.98 | | .14 | | < .001 | |
| PDS | | .20 | | .15 | | .191 | |
| Household Income | | -.37 | | .04 | | < .001 | |
| Parent Education | | -.17 | | .06 | | .008 | |
| ADI | | .01 | | .00 | | .002 | |
| Race/Ethnicity | |  | |  | |  | |
| Black | | -.13 | | .24 | | .584 | |
| Asian | | -1.04 | | .44 | | .019 | |
| Hispanic | | -.68 | | .18 | | < .001 | |
| Other | | .39 | | .22 | | .076 | |
| UPPS-P | |  | |  | |  | |
| Negative Urgency | | .24 | | .03 | | < .001 | |
| Positive Urgency | | .07 | | .03 | | .006 | |
| Lack of Premeditation | | .29 | | .03 | | < .001 | |
| Lack of Perseveration | | .12 | | .03 | | < .001 | |
| Sensation Seeking | | .02 | | .03 | | .485 | |
| Slope Effects | |  | |  | |  | |
| Sociodemographics | |  | |  | |  | |
| Sex (Female) | | .05 | | .06 | | .361 | |
| PDS | | .12 | | .06 | | .057 | |
| Household Income | | .02 | | .02 | | .259 | |
| Parent Education | | .04 | | .03 | | .124 | |
| ADI | | -.00 | | .00 | | .602 | |
| Race/Ethnicity | |  | |  | |  | |
| Black | | -.32 | | .10 | | .001 | |
| Asian | | .17 | | .18 | | .366 | |
| Hispanic | | .12 | | .08 | | .132 | |
| Other | | .16 | | .09 | | .202 | |
| UPPS-P | |  | |  | |  | |
| Negative Urgency | | -.04 | | .01 | | .001 | |
| Positive Urgency | | -.00 | | .01 | | .754 | |
| Lack of Premeditation | | -.04 | | .01 | | .003 | |
| Lack of Perseveration | | -.01 | | .01 | | .690 | |
| Sensation Seeking | | .00 | | .01 | | .695 | |

*Note.* CFI = Comparative Fit Index; TLI = Tucker-Lewis Index; RMSEA = Root Mean Square Error of Approximation; PDS = Pubertal Development Scale; ADI = Area Disadvantage Index.

**Table S3. Zero-order correlations between externalizing behaviors, impulsivity facets, and covariates.**

|  | **1** | **2** | **3** | **4** | **5** | **6** | **7** | **8** | **9** | **10** | **11** | **12** | **13** | **14** | **15** | **16** | **17** | **18** |
| --- | --- | --- | --- | --- | --- | --- | --- | --- | --- | --- | --- | --- | --- | --- | --- | --- | --- | --- |
| 1. **Ext. T1** | 1*** | 0.75*** | 0.69*** | 0.12*** | 0.03 | -0.18*** | -0.13*** | 0.1*** | -0.05*** | 0.06*** | -0.06*** | -0.01 | 0.04*** | 0.17*** | 0.14*** | 0.15*** | 0.12*** | 0.04*** |
| 1. **Ext. T2** |  | 1*** | 0.73*** | 0.1*** | 0.03* | -0.17*** | -0.12*** | 0.1*** | -0.04** | 0.05*** | -0.06*** | -0.01 | 0.04** | 0.15*** | 0.12*** | 0.14*** | 0.12*** | 0.04*** |
| 1. **Ext.T3** |  |  | 1*** | 0.09*** | 0.01 | -0.15*** | -0.1*** | 0.09*** | -0.04* | 0.02 | -0.04* | 0.01 | 0.05*** | 0.14*** | 0.13*** | 0.14*** | 0.12*** | 0.03 |
| 1. **Sex** |  |  |  | 1*** | -0.32*** | 0 | -0.01 | -0.01 | 0.02 | -0.02 | -0.01 | 0 | 0 | 0.08*** | 0.07*** | 0.12*** | 0.06*** | 0.13*** |
| 1. **Mean PDS** |  |  |  |  | 1*** | -0.23*** | -0.19*** | 0.16*** | -0.23*** | 0.28*** | -0.03 | 0.04** | 0.01 | -0.01 | 0.05*** | -0.05*** | -0.02 | -0.06*** |
| 1. **Income** |  |  |  |  |  | 1*** | 0.59*** | -0.42*** | 0.42*** | -0.37*** | 0.07*** | -0.22*** | -0.01 | -0.05*** | -0.15*** | 0.04** | -0.05*** | 0.07*** |
| 1. **Parent Education** |  |  |  |  |  |  | 1*** | -0.34*** | 0.34*** | -0.24*** | 0.11*** | -0.26*** | 0.01 | -0.05*** | -0.14*** | 0.04*** | -0.03* | 0.07*** |
| 1. **ADI** |  |  |  |  |  |  |  | 1*** | -0.17*** | 0.28*** | -0.19*** | 0.05*** | -0.02 | 0.04** | 0.1*** | -0.03* | 0.02 | -0.05*** |
| 1. **Race: White** |  |  |  |  |  |  |  |  | 1*** | -0.44*** | -0.15*** | -0.53*** | -0.36*** | -0.04** | -0.11*** | 0.06*** | 0 | 0.07*** |
| 1. **Black Race** |  |  |  |  |  |  |  |  |  | 1*** | -0.06*** | -0.21*** | -0.14*** | 0.05*** | 0.1*** | -0.05*** | -0.02 | -0.04** |
| 1. **Race: Asian** |  |  |  |  |  |  |  |  |  |  | 1*** | -0.07*** | -0.05*** | -0.02 | -0.01 | -0.01 | -0.01 | -0.02 |
| 1. **Race: Hispanic** |  |  |  |  |  |  |  |  |  |  |  | 1*** | -0.17*** | -0.01 | 0.03* | -0.04** | 0.01 | -0.05*** |
| 1. **Race: Other** |  |  |  |  |  |  |  |  |  |  |  |  | 1*** | 0.02 | 0.03 | 0.01 | 0.01 | 0 |
| 1. **Negative Urgency** |  |  |  |  |  |  |  |  |  |  |  |  |  | 1*** | 0.49*** | 0.16*** | 0.13*** | 0.14*** |
| 1. **Positive Urgency** |  |  |  |  |  |  |  |  |  |  |  |  |  |  |  | 0.21*** | 0.17*** | 0.19*** |
| 1. **L. of Premeditation** |  |  |  |  |  |  |  |  |  |  |  |  |  |  |  | 1*** | 0.45*** | 0.06*** |
| 1. **L. of Perseveration** |  |  |  |  |  |  |  |  |  |  |  |  |  |  |  |  | 1*** | -0.1*** |
| 1. **Sensation Seeking** |  |  |  |  |  |  |  |  |  |  |  |  |  |  |  |  |  | 1*** |
| **Note.** *p < .05, **p < .10, ***p < .001 | | | | | | | | | | | | | | | | | | |

**Table S4. Conditional Model Results After Removing Positive Urgency**

| **Parameter** | **Std. Est. (SE)** | ***p*** | **95% CI** |
| --- | --- | --- | --- |
| **Intercept Effects (T1)** |  |  |  |
| Sociodemographic Characteristics |  |  |  |
| Sex | -0.11 (0.01) | 0.000 | -0.13, -0.09 |
| PDS | 0.03 (0.01) | 0.003 | 0.01, 0.05 |
| Income | -0.18 (0.01) | 0.000 | -0.21, -0.15 |
| Parent Education | -0.04 (0.01) | 0.001 | -0.07, -0.02 |
| ADI | 0.03 (0.01) | 0.034 | 0.00, 0.05 |
| Race/Ethnicity |  |  |  |
| Black | -0.04 (0.01) | 0.003 | -0.06, -0.01 |
| Asian | -0.06 (0.01) | 0.000 | -0.08, -0.04 |
| Hispanic | -0.04 (0.01) | 0.000 | -0.06, -0.02 |
| Other | 0.02 (0.01) | 0.040 | 0.00, 0.04 |
| UPPS-P |  |  |  |
| Negative Urgency | 0.14 (0.01) | 0.000 | 0.12, 0.16 |
| Lack of Premeditation | 0.12 (0.01) | 0.000 | 0.10, 0.14 |
| Lack of Perseveration | 0.05 (0.01) | 0.000 | 0.03, 0.07 |
| Sensation Seeking | 0.02 (0.01) | 0.019 | 0.00, 0.04 |
| **Slope Effects (T1 – T3)** |  |  |  |
| Sociodemographic Characteristics |  |  |  |
| Sex | 0.04 (0.02) | 0.060 | 0.00, 0.09 |
| PDS | 0.02 (0.02) | 0.327 | -0.02, 0.07 |
| Income | 0.05 (0.03) | 0.089 | -0.01, 0.12 |
| Parent Education | 0.07 (0.03) | 0.015 | 0.01, 0.12 |
| ADI | 0.02 (0.02) | 0.488 | -0.03, 0.07 |
| Race/Ethnicity |  |  |  |
| Black | -0.06 (0.03) | 0.023 | -0.11, -0.01 |
| Asian | 0.03 (0.02) | 0.212 | -0.02, 0.08 |
| Hispanic | 0.02 (0.02) | 0.294 | -0.02, 0.06 |
| Other | 0.00 (0.02) | 0.866 | -0.04, 0.05 |
| UPPS-P |  |  |  |
| Negative Urgency | -0.09 (0.02) | 0.000 | -0.13, -0.04 |
| Lack of Premeditation | -0.07 (0.02) | 0.006 | -0.11, -0.02 |
| Lack of Perseveration | -0.01 (0.02) | 0.751 | -0.05, 0.04 |
| Sensation Seeking | 0.00 (0.02) | 0.923 | -0.04, 0.04 |
| *Note*. Model fit: χ(14) = 15.22, *p* = .363, CFI/TLI = 1.00/1.00, RMSEA = .003 (95% CI [.00, .009]). | | | |

**Table S5. Conditional Model Results After Removing Negative Urgency**

| **Parameter** | **Std. Est. (SE)** | ***p*** | **95% CI** |
| --- | --- | --- | --- |
| **Intercept Effects (T1)** |  |  |  |
| Sociodemographic Characteristics |  |  |  |
| Sex | -0.11 (0.01) | 0.000 | -0.13, -0.09 |
| PDS | 0.03 (0.01) | 0.006 | 0.01, 0.05 |
| Income | -0.18 (0.01) | 0.000 | -0.21, -0.15 |
| Parent Education | -0.04 (0.01) | 0.001 | -0.07, -0.02 |
| ADI | 0.02 (0.01) | 0.038 | 0.00, 0.05 |
| Race/Ethnicity |  |  |  |
| Black | -0.04 (0.01) | 0.003 | -0.06, -0.01 |
| Asian | -0.06 (0.01) | 0.000 | -0.09, -0.04 |
| Hispanic | -0.04 (0.01) | 0.000 | -0.06, -0.02 |
| Other | 0.02 (0.01) | 0.038 | 0.00, 0.04 |
| UPPS-P |  |  |  |
| Positive Urgency | 0.08 (0.01) | 0.000 | 0.06, 0.10 |
| Lack of Premeditation | 0.12 (0.01) | 0.000 | 0.10, 0.15 |
| Lack of Perseveration | 0.06 (0.01) | 0.000 | 0.03, 0.08 |
| Sensation Seeking | 0.03 (0.01) | 0.007 | 0.01, 0.05 |
| **Slope Effects (T1 – T3)** |  |  |  |
| Sociodemographic Characteristics |  |  |  |
| Sex | 0.05 (0.02) | 0.044 | 0.00, 0.09 |
| PDS | 0.02 (0.02) | 0.306 | -0.02, 0.07 |
| Income | 0.05 (0.03) | 0.090 | -0.01, 0.12 |
| Parent Education | 0.07 (0.03) | 0.016 | 0.01, 0.12 |
| ADI | 0.02 (0.02) | 0.487 | -0.03, 0.07 |
| Race/Ethnicity |  |  |  |
| Black | -0.06 (0.03) | 0.021 | -0.12, -0.01 |
| Asian | 0.03 (0.02) | 0.194 | -0.02, 0.08 |
| Hispanic | 0.02 (0.02) | 0.277 | -0.02, 0.06 |
| Other | 0.00 (0.02) | 0.887 | -0.04, 0.05 |
| UPPS-P |  |  |  |
| Positive Urgency | -0.04 (0.02) | 0.101 | -0.08, 0.01 |
| Lack of Premeditation | -0.07 (0.02) | 0.004 | -0.12, -0.02 |
| Lack of Perseveration | -0.01 (0.02) | 0.621 | -0.06, 0.03 |
| Sensation Seeking | 0.00 (0.02) | 0.899 | -0.05, 0.04 |
| *Note.* Model fit: χ(14) = 15.94, p = .317, CFI/TLI = 1.00/1.00, RMSEA = .003 (95% CI [.00, .01]). | | | |
